# Ferroptosis in Diabetic Cardiomyopathy and Atherosclerosis: Mechanisms and Clinical Prospects

**DOI:** 10.3390/ijms262110661

**Published:** 2025-11-01

**Authors:** Wenqiong Huang, Xumeng Han, Zongzhen Meng, Xiaoli Chen, Aiping Lyu, Kenneth C. P. Cheung

**Affiliations:** Hong Kong Traditional Chinese Medicine Phenome Research Center, Hong Kong Baptist University, Hong Kong, China

**Keywords:** ferroptosis, diabetic cardiomyopathy, atherosclerosis, iron metabolism, lipid peroxidation, GPX4, metabolic diseases

## Abstract

Ferroptosis, an iron-dependent form of regulated cell death, plays a pivotal role in the pathogenesis of cardiometabolic diseases (CMDs), particularly diabetic cardiomyopathy (DCM) and atherosclerosis (AS). This review comprehensively explores the metabolic pathways underlying ferroptosis, including dysregulation of iron, lipid, amino acid, and glucose metabolism, as well as involvement of the mevalonate pathway and key regulators such as NRF2 and p53. We analyze the cell type-specific mechanisms through which ferroptosis contributes to DCM and AS, driving myocardial dysfunction, plaque instability, and inflammatory amplification. Furthermore, we discuss emerging therapeutic strategies targeting ferroptosis, such as iron chelators, antioxidants, lipoxygenase inhibitors, ACSL4 inhibitors, nitroxides, and selenium supplements, which demonstrate potential in mitigating oxidative stress, restoring iron homeostasis, and suppressing inflammation. This review underscores the clinical relevance of targeting ferroptosis and highlights its promise as a novel therapeutic avenue for treating cardiometabolic diseases.

## 1. Introduction

Diabetic cardiomyopathy (DCM) and atherosclerosis (AS) are common manifestations of cardiometabolic diseases (CMDs). DCM is characterized by left ventricular diastolic and/or systolic dysfunction in diabetic patients, in the absence of hypertension, coronary artery disease, or valvular heart disease [1]. Its prevalence increases exponentially with diabetes duration, with approximately 30% of type 2 diabetes mellitus (T2DM) patients developing abnormal echocardiographic findings within 10 years of diagnosis. AS, on the other hand, underlies acute coronary syndromes and ischemic stroke, primarily contributing to ischemic heart disease (IHD) and ischemic stroke (IS) [2]. Plaque rupture-induced thrombotic events account for 54% of all cardiovascular deaths [3]. Despite the widespread use of conventional risk control strategies—such as antihypertensive agents, lipid-lowering drugs, antiplatelet therapy, and SGLT2 inhibitors—residual risk remains significant, implying the potential involvement of common downstream pathways that remain untargeted [4].

Cell death is a highly regulated process categorized into accidental cell death (ACD) and regulated cell death (RCD) [5]. RCD encompasses not only apoptosis but also multiple non-apoptotic forms, including autophagy, ferroptosis, necroptosis, and pyroptosis [5,6,7]. Ferroptosis, an iron-dependent novel type of RCD, was formally termed by Dixon et al. in 2012, though its inducers, such as erastin and Ras-selective lethal small molecules (RSLs), had been identified as early as 2003, accompanied by observations of distinct mitochondrial morphological changes [6,8,9,10]. The core mechanism of ferroptosis involves disruption of iron homeostasis, depletion of glutathione (GSH), and loss of glutathione peroxidase 4 (GPX4) activity, leading to excessive accumulation of lipid peroxides [6].

Notably, ferroptosis does not occur in isolation but interacts extensively with other RCD pathways [11,12,13]. Autophagy promotes iron release through the NCOA4-mediated ferritinophagy pathway, while the AMPK-mTOR signaling axis coordinately regulates the balance between these processes [14]. The apoptosis-related protein p53 can promote either apoptosis or ferroptosis by upregulating Bax and inhibiting SLC7A11 [15]. Activated caspase-3 can cleave and inactivate GPX4. Meanwhile, reactive oxygen species (ROS) generated by ferroptosis activate the NLRP3 inflammasome, thereby inducing pyroptosis [16]. Conversely, pores formed by the pyroptosis executioner protein GSDMD facilitate iron influx, creating a positive feedback loop [17]. Necroptosis and ferroptosis share a ROS amplification mechanism; in models such as myocardial ischemia–reperfusion injury, combined inhibition using agents such as Nec-1 and Fer-1 has shown synergistic protective effects [18].

Owing to its core “iron–lipid–ROS” triad, ferroptosis is closely linked to metabolic disturbances such as hyperglycemia, insulin resistance, iron overload, and chronic low-grade inflammation, which are hallmark features of diabetic conditions [19]. These metabolic abnormalities and oxidative stress collectively establish a microenvironment conducive to ferroptosis, thereby promoting cardiomyocyte death and atherosclerotic plaque formation. Thus, ferroptosis not only offers a novel molecular framework for understanding the pathogenesis of CMDs like DCM and AS but also highlights promising therapeutic directions for addressing residual risk through targeted interventions. This review comprehensively summarizes the mechanistic roles of ferroptosis in DCM and AS and discusses the clinical potential of targeting ferroptosis for preventing and treating these disorders, providing a theoretical foundation for developing new treatment strategies.

## 2. Metabolic Mechanisms of Ferroptosis

### 2.1. Iron Metabolism

**Figure 1:**
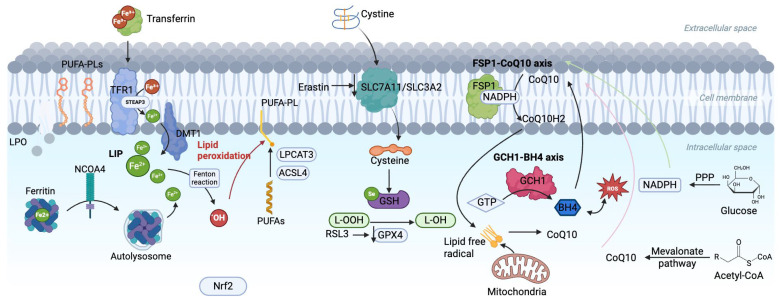
Core metabolic pathways and the multi-level defense network in ferroptosis.

As shown in Figure 1, iron, an essential trace element in the human body, plays a vital role in oxidative metabolism, enzymatic activity regulation, and electron transport [20]. Disorders of iron metabolism, such as iron overload and iron deficiency, can trigger various pathological processes, including ferroptosis [21]. Dietary iron is primarily derived from heme iron in animal products, predominantly in the ferrous form (Fe^2+^), and non-heme iron in plant sources, mainly in the ferric form (Fe^3+^) [22]. After absorption, Fe^3+^ binds to transferrin (TF) and enters cells via transferrin receptor (TFR1)-mediated endocytosis, where it is reduced to Fe^2+^ by six-transmembrane epithelial antigen of prostate 3 (STEAP3) and transported into the cytoplasm through divalent metal transporter 1 (DMT1) to form the labile iron pool (LIP), ensuring sufficient bioavailable iron for cellular functions [23,24,25]. On the other hand, TFR2, regulates ferritin expression and maintains homeostasis of iron metabolism throughout the body by adjusting the concentration of TF [26].

Excess iron not immediately utilized is stored in ferritin, composed of light (FTL1) and heavy (FTH1) chains, to prevent the generation of excessive ROS [27]. However, when Fe^2+^ accumulates excessively, it reacts with hydrogen peroxide (H_2_O_2_) through the Fenton reaction, producing hydroxyl radicals that drive lipid peroxidation and generate toxic lipid ROS, activating lipoxygenases (particularly 15-LOX) [27]. This lipid peroxidation further compromises membrane integrity, ultimately inducing ferroptosis [28]. IRP, an iron-sulfur cluster-containing protein, acts as a regulator of iron homeostasis, and IRP1 and IRP2 interact with iron-regulatory elements (IREs) in the untranslated regions (UTRs) of mRNAs encoded by proteins involved in iron metabolism (e.g., Ferritin, TFR) when iron levels are low, promoting the translation of TFR and increasing iron uptake, while inhibiting the translation of Ferritin and decreasing iron storage [29]. Additionally, ferritinophagy regulated by NCOA4 promotes the release iron from ferritin, exacerbating intracellular iron accumulation and oxidative damage [30].

**Figure 2:**
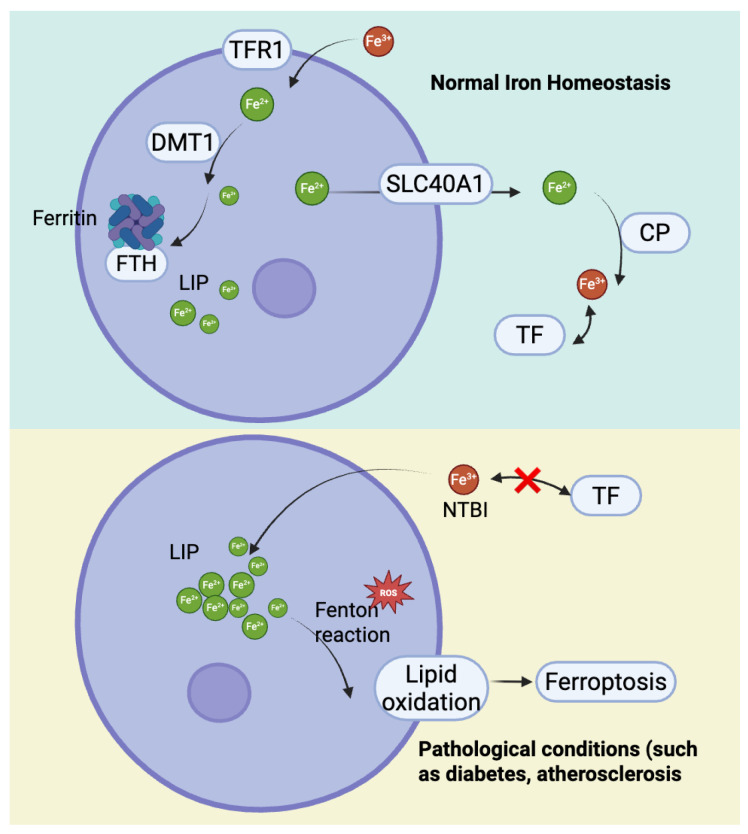
The mechanisms of cellular iron homeostasis and iron excretion and their imbalance under pathological conditions.

To maintain iron homeostasis, cells export excess iron via Ferroportin 1 (SLC40A1), while ceruloplasmin (CP) oxidizes Fe^2+^ to Fe^3+^ for TF transport [31,32]. In pathological conditions where iron exceeds TF’s binding capacity, non-transferrin-bound iron (NTBI) accumulates, leading to intracellular iron deposition [33]. These disruptions in iron metabolism not only increase cellular susceptibility to ferroptosis but also play a pivotal role in the development of CMDs (Figure 2) [34].

### 2.2. Lipid Metabolism

Ferroptosis is a form of iron-dependent cell death driven by lipid peroxidation that disrupts cell membrane integrity and leads to cell death [35]. The lipid bilayer of the cell membrane is rich in polyunsaturated fatty acids (PUFAs), which are more susceptible to peroxidation than monounsaturated fatty acids (MUFAs). This is due to the weaker carbon-carbon double bonds in PUFAs such as arachidonic acid (AA) and adrenergic acid (AdA) [36]. The biosynthesis of PUFAs is a key step in ferroptosis [37]. Enzymes such as acyl-CoA synthetase long-chain family member 4 (ACSL4) catalyze the binding of long-chain PUFAs to coenzyme A (CoA) to form PUFA-CoA [38]. Lysophosphatidylcholine acyltransferase 3 (LPCAT3) then re-esterifies PUFA-CoA into phospholipids such as PUFA-phosphatidylethanolamine (PUFA-PE) and PUFA-phosphatidylcholine (PUFA-PC). These phospholipids are major drivers of ferroptosis and can also be recognized by immune cells, triggering inflammation (Figure 1) [36].

Lipid peroxidation can occur by both non-enzymatic and enzymatic mechanisms [39]. In the non-enzymatic mechanism, NADPH-cytochrome P450 reductase (POR) and NADH-cytochrome b5 reductase (CYB5R1) transfer electrons from NAD(P)H to oxygen, producing hydrogen peroxide (H_2_O_2_) [40]. Superoxide anions (O_2_^•−^) produced by mitochondrial respiratory chain dysfunction can be converted to H_2_O_2_ by superoxide dismutase (SOD) [39]. In the presence of iron, H_2_O_2_undergoes the Fenton reaction to produce hydroxyl radicals (-OH), which then abstract hydrogen atoms from the bis-allylic positions of PUFA-containing phospholipids. This produces lipid radicals (PL^•^), which react with oxygen to form peroxyl radicals (PLOO^•^) [41]. These PLOO^•^ then interact with another PUFA phospholipid to form lipid hydroperoxides (LOOH) and a new PLOO^•^, continuing the lipid peroxidation. This chain reaction is eventually terminated when two PLOO^•^ radicals interact to form non-radical products [39]. In the enzymatic mechanism, non-heme iron-containing dioxygenases such as lipoxygenases (LOX) and cyclooxygenase 2 (COX2) catalyze the oxidation of PUFA phospholipids, generating lipid peroxides such as malondialdehyde (MDA) and 4-hydroxynonenal (4-HNE), which accelerate ferroptosis and compromise membrane integrity [41].

In addition, lipid metabolism plays a critical role in the regulation of ferroptosis. MUFAs are converted to acyl-CoA esters by acyl-CoA synthetase long-chain family member 3 (ACSL3) [42]. These MUFAs are incorporated into the cell membrane by stearoyl-CoA desaturase 1 (SCD1), thereby enhancing antioxidant capacity and preventing ferroptosis [43]. In contrast, long-chain saturated fatty acids (SFA) are reduced to fatty alcohols (FAL) by peroxisomal fatty acyl-CoA reductase 1 (FAR1), generating polyunsaturated ether phospholipids (PUFA-ePL) that serve as substrates for lipid peroxidation and promote ferroptosis [44].

### 2.3. Amino Acid Metabolism

Amino acid metabolism plays an important role in the regulation of iron death, in which GSH, as a major antioxidant molecule, can reduce oxidative damage and inhibit iron death by converting lipid hydroperoxides to less toxic lipids and alcohols [45]. The cycle of GSH is mediated by glutathione reductase (GR), which maintains intracellular antioxidant homeostasis by reducing oxidized glutathione (GSSG) to reduced GSH with the use of NADPH [46]. GSH synthesis requires γ-glutamylcysteine synthetase (γ-GCS), which catalyzes the formation of γ-glutamylcysteine from glutamate and cysteine, which binds to glycine via glutathione synthetase (GS) to form the tripeptide GSH [47].

The source of cysteine can be exogenous uptake mediated by system Xc^−^ and the transsulfuration pathway [48]. System Xc^−^ consists of solute carrier family 3 member 2 (SLC3A2) and solute carrier family 7 member 11 (SLC7A11) and transports extracellular cystine into the cell where it is reduced to cysteine [49]. The transsulfuration pathway maintains endogenous cysteine levels by metabolizing methionine and homocysteine [50]. Specifically, methionine is converted to homocysteine (Hcy) via one-carbon metabolism and then catalyzed by cystathionine beta-synthase (CBS) to cysteine in the presence of serine [51]. In addition, cystathionine γ-cleaving enzyme (CSE) breaks down cysteine to release Hcy, which can be methylated to produce methionine, creating a dynamic equilibrium [51]. The activity of the Xc system is regulated by nuclear factor erythroid 2-related factor 2 (NRF2) and p53 [52]. NRF2 upregulates SLC7A11 to enhance antioxidant defenses, while p53 inhibits its expression, thereby reducing cysteine uptake and promoting the accumulation of lipid peroxidation, which exacerbates iron metamorphosis [53]. Under pathological conditions such as atherosclerosis, oxidative stress driven by heme and free iron released by hemorrhage within plaques is an important inducer for consuming the intracellular GSH reservoir and disrupting the thiol REDOX balance. This further intensifies the cells’ reliance on cystine uptake and GSH synthesis systems, making them more vulnerable to ferroptosis stress [54,55].

In addition, glutamate plays a critical role in GSH synthesis. Glutamate is produced by glutaminase (GLS) through the enzymatic catabolism of glutamine and is transported into the cell via transporters such as SLC38A1 and SLC1A5 [56]. Glutamate combines with cysteine and glycine to form GSH, which maintains cellular redox balance [57]. In addition, α-ketoglutarate from glutamate metabolism enters the tricarboxylic acid (TCA) cycle and participate in glucose metabolism, while ammonia is detoxified via the urea cycle [58,59].

GPX4 is a selenium-dependent antioxidant enzyme that acts by depleting two GSH molecules, reducing lipid peroxides to non-toxic lipohydrols, inhibiting accumulation of lipid peroxides, and preventing iron death [60]. Erastin reduces GSH levels and GPX4 activity by inhibiting Xc^−^, while RSL3 directly inhibits GPX4, both of which promote iron death (Figure 1) [60].

### 2.4. Glucose Metabolism

Glucose is the primary energy source for cells, and its metabolism plays a critical intermediary role in ferroptosis by providing key metabolites that influence redox balance and lipid peroxidation, which are essential for ferroptosis [61]. After glucose enters the cell through glycolysis, it is initially broken down by hexokinase (HK) into glucose-6-phosphate (G6P), which is then converted into fructose-6-phosphate (F6P) by G6P isomerase and ultimately broken down into pyruvate [62]. Part of the pyruvate is converted to lactate by lactate dehydrogenase, and lactate increases the levels of NADH, NADPH, and GSH, thereby inhibiting ferroptosis (Figure 1) [63]. The remaining pyruvate enters the mitochondria, where it undergoes the TCA cycle, generating NADH and FADH, which are essential for cellular biosynthesis and energy production [64]. These molecules participate in mitochondrial oxidative phosphorylation to maintain ATP production and control ROS levels [65]. However, disruption of mitochondrial glucose metabolism leads to excessive ROS accumulation, which, if not neutralized by NADPH-dependent pathways, triggers lipid peroxidation and results in ferroptosis [61].

Moreover, ATP and NADH generated from glycolysis are converted through the pentose phosphate pathway (PPP) into NADPH and ribose-5-phosphate [66]. NADPH reduces oxidized GSSG to its reduced form as GSH, maintaining GPX4 activity and inhibiting ferroptosis [67]. Glucose-6-phosphate dehydrogenase (G6PD), the rate-limiting enzyme in the PPP, plays a crucial role in this process [68]. Abnormal expression of G6PD disrupts the metabolism of NADPH and GSH, leading to redox imbalance in the cell, which in turn induces ferroptosis [51].

Impaired glycolysis not only affects the function of the transsulfuration pathway but also inhibits cystine uptake mediated by the SLC7A11-SLC3A2 (Xc^−^ antiporter system), reducing intracellular cysteine levels, which are essential for GSH synthesis and further influencing ferroptosis [61]. Dysregulation of glucose metabolism also manifests as excessive glucose, leading to an accumulation of acetyl-CoA, which promotes fatty acid synthesis and increases PUFAs, thereby exacerbating lipid peroxidation [69]. On the other hand, glucose starvation activates the AMPK pathway, reducing fatty acid synthesis and inhibiting lipid production [67]. Thus, glucose metabolism dysregulation disrupts cellular energy balance and redox homeostasis, ultimately affecting the occurrence of ferroptosis [61].

### 2.5. The Mevalonate Pathway

The mevalonate pathway transforms acetyl-CoA into acetylacetyl-CoA under the action of HMG-CoA synthase, which is subsequently converted to 3-hydroxy-3-methylglutaryl-CoA (HMG-CoA) [70]. HMG-CoA is reduced to mevalonate by HMG-CoA reductase (HMGCR), which undergoes phosphorylation and decarboxylation to form isopentenyl pyrophosphate (IPP) and dimethylallyl pyrophosphate (DMAPP) [71]. These intermediates are further converted to farnesyl pyrophosphate (FPP) and geranylgeranyl pyrophosphate (GGPP), which are essential for synthesizing cholesterol, coenzyme Q10 (CoQ10), and other vital biomolecules, playing crucial roles in protein prenylation and cellular signaling [72]. FPP is catalyzed by squalene synthase (SQS) to form squalene, which is then converted into 2,3-epoxysqualene by squalene epoxidase (SQLE), involved in redox reactions and immune regulation [73].

CoQ10, a key molecule in the mitochondrial inner membrane, undergoes reversible redox cycling between its oxidized quinone and reduced quinol forms, which is crucial for oxidative phosphorylation [74]. It facilitates the electron transport chain (ETC) by transferring electrons from Complex I (NADH dehydrogenase) and Complex II (succinate dehydrogenase) to Complex III (cytochrome c reductase), driving ATP production to meet cellular energy demands [75]. Additionally, as a lipid-soluble antioxidant, reduced CoQ10 (CoQH_2_) directly scavenges and neutralizes ROS, mitigating lipid peroxidation and protecting mitochondrial membranes and other cellular structures [76]. Disruption of the mevalonate pathway impairs antioxidant defenses, increasing oxidative stress, lipid peroxidation, and ferritin deposition, thereby promoting ferroptosis [77]. Statins, which block HMGCR to lower cholesterol, also reduce CoQ10 levels, impair membrane integrity, and interfere with cellular signaling, hindering GPX4 translation and exacerbating ferroptosis [78]. Furthermore, the type 3 ferroptosis inducer, Fin56, also promotes ferroptosis partly by depleting CoQ10 [79].

The antioxidant activity of CoQ10 depends on the reducing power provided by NADPH, which is generated through mitochondrial glucose metabolism [80]. Ferroptosis suppressor protein 1 (FSP1), an independent ferroptosis inhibitor, is myristoylated and targeted to the plasma membrane, where it utilizes NADPH to reduce CoQ10 to its active form, thereby enhancing antioxidant defenses and protecting plasma membrane lipids [77]. However, when glucose metabolism is impaired, NADPH levels drop, limiting FSP1 activity, weakening CoQ10’s antioxidant capacity, and promoting ferroptosis through increased lipid peroxidation (Figure 1) [74].

### 2.6. Other Pathways

NRF2 is a key transcription factor that regulates various genes involved in iron metabolism and oxidative stress, such as glutamate-cysteine ligase catalytic subunit (GCLC), heme oxygenase 1 (HO-1), and NAD(P)H:quinone oxidoreductase 1 (NQO1) [81]. These pathways help reduce the accumulation of free radicals and ROS within cells, thereby alleviating oxidative stress [82]. Under normal physiological conditions, NRF2 is maintained at low levels, bound to Keap1 in the cytoplasm. When exposed to oxidative stress or inflammatory stimuli, NRF2 dissociates from Keap1 and translocates to the nucleus, where it binds to antioxidant response elements (AREs) to initiate gene expression and activate downstream antioxidant and anti-inflammatory mechanisms [83]. Among these, activation of the NRF2-GCLC axis promotes the synthesis of GSH, which effectively inhibits lipid peroxidation [81]. Meanwhile, the NRF2-HO-1 axis degrades heme to produce Fe^2+^, bilirubin, and carbon monoxide (CO), with Fe^2+^ playing a crucial role in iron metabolism, while bilirubin and low concentrations of CO exhibit antioxidant properties that help maintain cellular redox balance [82]. Additionally, the NRF2-NQO1 axis reduces oxidized quinone compounds, such as CoQ10 and vitamin E, thereby decreasing oxidative damage and providing anti-inflammatory effects [82]. Studies have shown that NRF2 regulates the key ferroptosis-related genes SLC7A11 and GPX4, improving the dysregulated levels of iron and the accumulation of ROS, reactive nitrogen species (RNS), and reactive lipid species (RLS) [84,85]. Meanwhile, the Hippo pathway interacted with NRF2 to regulate YAP/TAZ activity and inhibit the expression of ACSL4 and LPCAT3 [81]. Therefore, NRF2 plays a crucial role in protecting cells from ferroptosis.

p53 is a critical tumor suppressor that plays a significant role in the metabolic regulation of ferroptosis. Studies have shown that p53 can inhibit the expression of SLC7A11 on the plasma membrane, blocking cystine uptake, leading to decreased synthesis of GSH and GPX4, thereby exacerbating lipid peroxidation and promoting ferroptosis [86]. Further research reveals that p53 interacts with the deubiquitinase USP7, inducing its nuclear translocation and removing the monoubiquitination of histone H2B at lysine 120 (H2Bub1), thereby reducing the expression of SLC7A11 [87]. In lipid metabolism, p53 activates spermidine/spermine N1-acetyltransferase 1 (SAT1), which catalyzes the N1-acetylation of spermidine and spermine to produce putrescine. This upregulates the expression of lipoxygenases ALOX15 and ALOX12, enhancing the activity of ACSL4-LPCAT3 pathway, promoting PUFAs peroxidation, leading to mitochondrial dysfunction and ferroptosis [88]. Additionally, p53 further potentiates ferroptosis by regulating several metabolic factors, including glutaminase 2 (GLS2), cyclooxygenase-2 (PTGS2), and ferredoxin reductase (FDXR), as well as specific non-coding RNAs. Interestingly, p53 also inhibits ferroptosis through specific signaling pathways, such as regulating dipeptidyl peptidase-4 (DPP4) and PINK1/Parkin-mediated mitophagy [89]. For example, p53 binds to DPP4, preventing its interaction with NADPH oxidase 1 (NOX1), thereby reducing lipid peroxidation levels. Additionally, studies have shown that DPP4 inhibitors can effectively suppress ferroptosis in p53-deficient colorectal cancer cells, further emphasizing the critical role of the p53-DPP4 interaction in ferroptosis regulation [90].

## 3. Ferroptosis in Diabetic Cardiomyopathy

DCM is an independent cardiovascular complication of diabetes, characterized by myocardial hypertrophy, fibrosis, and impaired systolic/diastolic function, which can progress to heart failure. DCM may develop in diabetic patients even in the absence of coronary artery disease or hypertension, and its prevalence increases with the duration of diabetes [91].

### 3.1. Mechanisms of Ferroptosis in DCM

**Figure 3:**
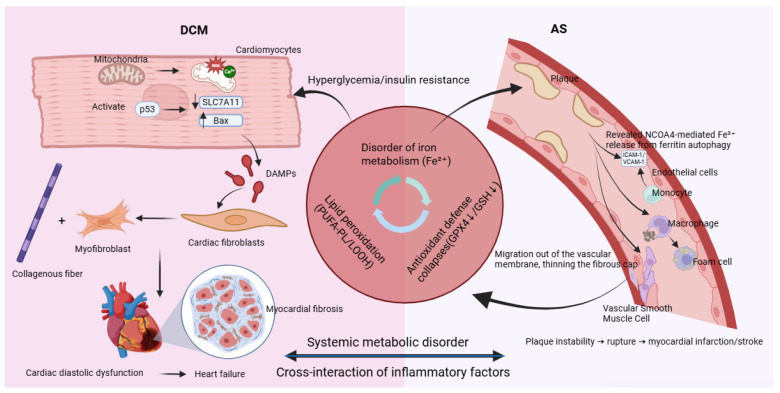
Ferroptosis: a common molecular bridge connecting diabetic cardiomyopathy and atherosclerosis.

The development and progression of DCM are closely linked to ferroptosis in cardiomyocytes—a vicious cycle driven by hyperglycemia and reinforced through multiple interconnected mechanisms. The process begins with the abnormal accumulation of free divalent iron (Fe^2+^) in cardiomyocytes induced by hyperglycemia. High glucose levels promote the formation of advanced glycation end products (AGEs), which activate NOX4 via the receptor for AGEs (RAGE), inducing oxidative stress. This results in upregulation of iron import proteins (DMT1 and TFR1) and suppression of the iron exporter SLC40A1, leading to increased cellular iron uptake and impaired iron efflux. Consequently, levels of Fe^2+^ within the oxidative active NTBI and LIP rise significantly, setting the stage for ferroptosis, as shown in Figure 3 [92,93,94].

Simultaneously, the core antioxidant defense system mediated by GPX4 is compromised through two major mechanisms. AGEs epigenetically suppress GPX4 transcription via the PKCβ-Sp1 signaling pathway. In addition, high glucose-induced molecules such as miR-214-3p directly target the 3′ UTR of GPX4 mRNA and inhibit its translation. The loss of GPX4 activity impairs the clearance of lipid peroxides, markedly exacerbating lipid peroxidation damage [92].

Mitochondria play a central role in the execution of ferroptosis. Accumulated Fe^2+^ promotes abnormal opening of the mitochondrial permeability transition pore (mPTP), disrupting calcium (Ca^2+^) coupling between mitochondria and the sarcoplasmic reticulum. This leads to aberrant Ca^2+^ transients and defective energy metabolism, impairing cardiomyocyte relaxation function—an early characteristic of DCM that often precedes systolic decline [92,95].

Ultimately, ferroptotic cardiomyocytes rupture and release damage-associated molecular patterns (DAMPs) such as HMGB1 and IL-1α. These activate classical inflammatory pathways including TLR4/NF-κB, recruiting immune cells and promoting persistent activation of cardiac fibroblasts. These fibroblasts differentiate into activated myofibroblasts that excessively produce collagen, leading to myocardial fibrosis. An increased collagen I/III ratio further reduces myocardial compliance, driving the heart toward stiffness and failure. In diabetes, hyperglycemia disrupts iron metabolism and inhibits GPX4, triggering ferroptosis. In turn, ferroptosis amplifies myocardial inflammation, fibrosis, and dysfunction through pro-inflammatory and pro-fibrotic signals, establishing a critical pathogenic axis in DCM [96].

### 3.2. Preclinical Evidence

A growing body of preclinical evidence supports a central role for ferroptosis in DCM pathogenesis. Typical hallmarks of ferroptosis—including decreased GPX4 activity, increased ACSL4 expression, and elevated lipid peroxidation products—have been consistently observed across models.

Functional studies show that inhibition of ferroptosis yields beneficial effects. In both T1DM (STZ-induced) and T2DM (db/db) mouse models, treatment with the ferroptosis-specific inhibitor Ferrostatin-1 for four weeks effectively improved cardiac diastolic dysfunction (18% reduction in E/e′ ratio) and significantly attenuated myocardial fibrosis (34% decrease in collagen volume fraction, CVF). Genetic evidence further supports this: pancreatic β-cell and cardiomyocyte dual-specific GPX4 knockout mice (GPX4β-cMHC-Cre) exhibited exacerbated myocardial hypertrophy and diastolic dysfunction, highlighting how intrinsic antioxidant defects in cardiomyocytes and systemic metabolic disturbances synergistically drive disease progression [97,98].

## 4. Ferroptosis in Atherosclerosis

AS is a major macrovascular complication in diabetes, characterized by lipid deposition, chronic inflammation, and plaque formation within the arterial wall. Plaque instability and rupture can lead to acute cardiovascular events such as myocardial infarction and stroke [99].

### 4.1. Vascular Iron Origin and Homeostasis in Atherosclerosis

Atherosclerotic plaques accumulate redox-active iron primarily through intraplaque hemorrhage (IPH). Erythrocyte extravasation from fragile neovessels releases hemoglobin and heme, which are cleared via macrophage scavenger receptors (CD163 for hemoglobin–haptoglobin, CD91/LRP1 for heme–hemopexin). Heme oxygenase-1 (HO-1) degrades heme, releasing Fe^2+^—a potent catalyst of lipid peroxidation—while generating bilirubin and CO, which exert antioxidant effects. Ferritin, especially the ferroxidase-active heavy chain (FTH1), serves as a key cytoprotective buffer in endothelial cells and macrophages by sequestering iron and limiting oxidative injury. However, in advanced hemorrhagic lesions, this system can be overwhelmed, leading to iron overload, enhanced lipid peroxidation, and plaque destabilization. Intraplaque hemorrhage (IPH) is a dominant, intrinsic source of redox-active iron within human atherosclerotic lesions. Fragile neovessels in the plaque shoulder and core permit erythrocyte extravasation; subsequent hemolysis releases hemoglobin and heme, seeding iron deposition and catalyzing lipid oxidation. Pathology studies in human coronary and carotid arteries consistently link IPH to necrotic-core expansion, fibrous-cap thinning, and rupture-prone phenotypes [100,101,102,103,104,105].

### 4.2. The Dual Role of Nitric Oxide in Atherosclerotic Ferroptosis

Endothelial nitric oxide (NO), produced by eNOS, functions as a *chain-breaking antioxidant* that inhibits lipid peroxidation by scavenging lipid peroxyl radicals. However, under oxidative stress conditions (e.g., diabetes, hyperlipidemia), eNOS becomes uncoupled due to BH4 depletion and increased superoxide (O_2_^•−^). Uncoupled eNOS produces O_2_^•−^, which reacts with NO to form *peroxynitrite (ONOO^−^)*—a potent pro-oxidant that promotes lipid and protein oxidation. Thus, NO’s role shifts from anti- to pro-ferroptotic depending on the redox milieu. Restoring NO bioavailability—via BH4 supplementation, statins, or lifestyle interventions—represents a potential strategy to counteract ferroptosis in atherosclerosis [106,107,108,109,110].

### 4.3. Cell Type-Specific Mechanisms

As a chronic inflammatory disease, atherosclerosis involves ferroptosis across multiple vascular cell types—endothelial cells, macrophages, and vascular smooth muscle cells (VSMCs)—each contributing to disease initiation, progression, and ultimate plaque rupture [111].

As shown in Figure 2, in endothelial cells, pathogenic factors such as disturbed flow and high glucose-induced AGEs trigger NCOA4-mediated ferritinophagy, resulting in substantial release of free Fe^2+^. Iron overload upregulates NOX1 and LOX-1, increasing ROS and promoting expression of adhesion molecules ICAM-1 and VCAM-1. This enhances monocyte adhesion and recruitment to the endothelium, initiating atherosclerotic plaque formation [112].

In macrophages, uptake of ox-LDL via CD36 activates the Src-ERK1/2 pathway, upregulating ACSL4 and promoting synthesis of polyunsaturated fatty acid phospholipids (PUFA-PLs), which serve as substrates for lipid peroxidation. Meanwhile, oxidative stress-activated p53 inhibits expression of the cystine transporter SLC7A11, GSH and inactivating GPX4. These pathways synergize to induce robust ferroptosis. Dying macrophages release lipid contents, forming and expanding the necrotic core—a key feature of plaque instability [111].

In VSMCs, factors such as PDGF-BB activate the STAT3 pathway, upregulating TFR1 and increasing iron uptake, leading to ferroptosis. Ferroptosis promotes a phenotypic switch in VSMCs from a contractile (α-SMA^+^) to a synthetic (OPN^+^) state. This transition mediates VSMC migration from the medial layer, reduces collagen secretion, and thins the fibrous cap, thereby increasing plaque vulnerability [112].

Through cell-specific mechanisms, ferroptosis contributes critically to atherosclerosis: endothelial ferroptosis initiates inflammatory recruitment; macrophage ferroptosis enlarges the necrotic core; and VSMC ferroptosis weakens fibrous cap integrity. Together, these processes promote inflammation, lipid accumulation, and plaque destabilization, elevating the risk of acute ischemic events. Targeting ferroptosis has thus emerged as a promising therapeutic strategy for atherosclerotic cardiovascular disease.

### 4.4. Ferroptosis and Plaque Stability

Ferroptosis significantly influences atherosclerotic plaque stability, which depends on fibrous cap thickness, inflammation extent, and necrotic core size. By promoting endothelial dysfunction, enhancing inflammation, increasing cell death, and impairing fibrous cap integrity, ferroptosis drives plaques toward a vulnerable state [113].

Clinical evidence from human carotid endarterectomy specimens shows that ferroptosis area (identified by TUNEL^+^/PTGS2^+^ staining) correlates negatively with fibrous cap thickness. Patients with high ACSL4 expression had a 3.4-fold higher risk of ipsilateral ischemic events within two years, consistent with molecular features of unstable plaques such as SLC7A11 downregulation, reduced GPX4 activity, and ACSL4 upregulation [114].

Preclinical studies demonstrate that targeting ferroptosis can stabilize plaques. Treatment with the ACSL4 inhibitor AS-252424 for eight weeks significantly reduced plaque area, shrunk the necrotic core, and thickened the fibrous cap [115].

Furthermore, the application of ^18^F-PUFA PET/CT imaging allows non-invasive in vivo detection of plaque lipid peroxidation, offering a translational tool for assessing plaque vulnerability and monitoring anti-ferroptosis therapies [116]. As a key driver of plaque destabilization, ferroptosis represents a promising therapeutic target for stabilizing vulnerable plaques and preventing acute cardiovascular events.

## 5. Therapeutic Strategies for Targeting Ferroptosis in Cardiometabolic Diseases

Ferroptosis inhibitors are emerging as critical therapeutic agents that mitigate or prevent cell damage caused by ferroptosis through diverse mechanisms. This section outlines the main categories of ferroptosis inhibitors, their representative drugs and their mechanisms of action are shown in Table 1.

### 5.1. Iron Chelators

Iron chelators, such as DFO and Deferiprone (DFP), are pivotal in managing iron overload disorders by forming stable complexes with iron ions, thereby reducing intracellular free iron levels [117]. This mechanism is crucial in inhibiting iron-dependent oxidative stress and lipid peroxidation, which are key drivers of cellular damage and disease progression [118]. Additionally, iron chelators mitigate inflammation by reducing ROS and modulate immune responses by influencing the activity and polarization of immune cells, including T cells, B cells, and macrophages [119].

### 5.2. Antioxidants

Antioxidants, such as Ferrostatin-1 and Liproxstatin-1, play a critical role in mitigating oxidative stress and cellular damage associated with ROS and RNS. These compounds function by scavenging ROS, which are predominantly produced as byproducts of cellular metabolism, and preventing the formation of additional ROS through the inhibition of the Fenton reaction [120]. Moreover, a study demonstrated that Liproxstatin-1 not only inhibited mitochondrial lipid peroxidation but also restored the expression of GSH, GPX4, and ferroptosis suppressor protein 1 in oligodendrocytes [121].

### 5.3. Lipoxygenase (LOX) Inhibitors

LOX inhibitors, including Zileuton, AA861, PD146176, and Baicalin, are compounds that play a significant role in modulating ferroptosis [122]. These inhibitors function by inhibiting specific LOX enzymes, which are key players in the peroxidation of PUFAs, such as arachidonic acid and linoleic acid [123]. By curbing the enzymatic activity of LOX, these inhibitors reduce the generation of ROS and suppress lipoxygenase activity, thereby lowering intracellular ROS levels and mitigating lipid peroxidation, which is central to ferroptotic cell death [124]. This inhibition is crucial in managing the oxidative stress associated with ferroptosis. As a result, LOX inhibitors can potentially modulate the ferroptotic pathway and provide therapeutic benefits in diseases where ferroptosis plays a pathological role, making them valuable tools in the management of conditions associated with this form of cell death.

### 5.4. ACSL4 Inhibitors

ACSL4 inhibitors play a crucial role in the modulation of ferroptosis. These inhibitors function by targeting the enzyme ACSL4, which is involved in the activation of PUFAs. By inhibiting ACSL4 activity, the generation of lipid peroxidation substrates is decreased, subsequently reducing the propagation of ferroptosis [115]. This mechanism of action has been identified as a potential therapeutic strategy in diseases associated with ferroptosis, including neurodegenerative disorders and cancer [125]. Recent research has highlighted the specificity of ACSL4 inhibitors, such as AS-252424 (AS), which has been shown to bind directly to ACSL4 and inhibit its enzymatic activity, thereby suppressing lipid peroxidation and ferroptosis in both human and mouse cells [115].

### 5.5. Nitroxides

Nitroxides, such as TEMPO, have emerged as potent catalytic inhibitors of ferroptosis. Nitroxides such as TEMPO act as stable free radicals that catalytically scavenge reactive oxygen species (ROS) by undergoing reversible redox cycling between nitroxide and hydroxylamine forms. They inhibit the Fenton reaction by competing with H_2_O_2_ for Fe^2+^, thereby reducing •OH generation and suppressing lipid peroxidation. This dual mechanism of direct radical scavenging and iron-ion sequestration makes them highly effective in cellular and animal models of ferroptosis [126,127].

### 5.6. Selenium Supplements

Selenium supplements, including Selenium, Methylselenocysteine, and Selenomethionine, are recognized for their role in ferroptosis inhibition through the enhancement of GPX4 abundance and the subsequent improvement in the clearance capacity of lipid peroxides [122]. GPX4 is a selenoenzyme that utilizes GSH to reduce lipid hydroperoxides, thereby preventing the propagation of lipid peroxidation, a hallmark of ferroptosis. The supplementation of selenium has been shown to increase GPX4 activity, which is crucial in counteracting the oxidative stress associated with ferroptosis [128].

### 5.7. Integrated Therapeutic Strategies Targeting Ferroptosis in DCM

#### 5.7.1. Regulation of Oxidative Stress and Lipid Peroxidation

In diabetic cardiomyopathy, oxidative stress and lipid peroxidation induced by chronic hyperglycemia represent one of the core mechanisms leading to myocardial structural and functional impairment [129].

The classical ferroptosis inhibitors Ferrostatin-1 and Liproxstatin-1 have demonstrated significant efficacy in preclinical DCM models. In both STZ-induced T1DM and db/db T2DM mouse models, Ferrostatin-1 treatment improved cardiac diastolic function and attenuated myocardial fibrosis by restoring GPX4 expression and inhibiting lipid peroxidation [130,131]. Similarly, Liproxstatin-1 alleviated high glucose-induced cardiomyocyte death and mitochondrial damage by upregulating GPX4 and SLC7A11 expression [132].

Furthermore, LOX inhibitors such as Baicalin can mitigate myocardial ischemia–reperfusion injury and improve cardiac function parameters (e.g., LVEF, LVSP, and -dp/dt max) by inhibiting the chain reaction of lipid peroxidation [133]. These studies collectively indicate that antioxidant therapy targeting ferroptosis can effectively alleviate myocardial injury and structural remodeling in DCM.

#### 5.7.2. Regulation of Iron Metabolism

Iron overload is a widely recognized pathogenic factor in DCM. It promotes the generation of ROS via the Fenton reaction, thereby inducing ferroptosis in cardiomyocytes. The classic chelators DFO and DFP have shown significant cardioprotective effects. In models of iron overload-related cardiac dysfunction, monotherapy with DFO or combination therapy with DFP significantly reduced serum non-transferrin-bound iron (NTBI), decreased myocardial iron deposition, and improved left ventricular function [134,135].

In DCM models, DFO intervention upregulated HIF-1α expression, suppressed the expression of ferroptosis-related proteins (e.g., ACSL4, PTGS2), alleviated myocardial fibrosis, and improved myocardial compliance [136].

### 5.8. Integrated Therapeutic Strategies Targeting Ferroptosis in AS

#### 5.8.1. Inhibition of Ferroptosis in Vascular Endothelium and Macrophages

In AS, therapeutic goals are centered on preserving endothelial function, reducing macrophage ferroptosis in the necrotic core, and maintaining plaque stability.

AS-252424, a selective ACSL4 inhibitor, significantly reduced plaque area, shrunk the necrotic core, increased fibrous cap thickness, and markedly decreased the plaque vulnerability index after 8 weeks of intervention in ApoE^−/−^ mouse models. Its mechanisms include inhibiting lipid peroxidation, reducing macrophage ferroptosis and the release of inflammatory factors, and enhancing plaque stability [115].

To enhance specificity and safety, endothelial-targeted nanotherapies have been developed. For instance, DFO-loaded PLGA-VCAM-1 nanoparticles exhibited an 8.7-fold higher enrichment in plaque areas, significantly reduced local MDA levels, restored GPX4 activity, and decreased macrophage infiltration without systemic interference with iron metabolism [137].

#### 5.8.2. Molecular Imaging and Biomarker-Guided Therapy

The translation of anti-ferroptosis therapies into the clinic is critically dependent on the development of companion diagnostics for patient stratification and treatment monitoring. Emerging theranostic platforms are now demonstrating the direct feasibility of this approach. A recent study developed a folate receptor-targeted polydopamine nanoplatform (FPLG NPs) that concurrently serves as a PET/CT imaging agent and a ferroptosis inhibitor. This system achieved targeted accumulation in atherosclerotic plaques, as visualized by PET/CT, and subsequently attenuated foam cell ferroptosis by scavenging ROS and upregulating GPX4 and NRF2 activity, leading to a >40% reduction in plaque area in ApoE^−/−^ mice [138]. This work provides a compelling proof-of-concept for image-guided, targeted anti-ferroptosis therapy.

Complementing imaging, biomarker models offer a complementary strategy for risk assessment. A combined model based on serum NTBI, ACSL4 mRNA, and GPX4 activity demonstrated excellent predictive performance (AUC = 0.89) for the risk of ischemic events within 2 years in AS patients [116]. This biomarker panel not only aids in risk prognostication but also provides a mechanistic basis for selecting and monitoring targeted interventions.

Together, these advances in molecular imaging and biomarker science are paving the way for a personalized treatment strategy in atherosclerotic cardiovascular disease, enabling the right anti-ferroptosis therapy to be delivered to the right patient at the right time.

## 6. Conclusions and Perspectives

CMDs represent a significant and growing threat to global health due to their high morbidity and mortality. Ferroptosis, an iron-dependent form of regulated cell death, has emerged as a critical pathological mechanism linking various CMDs through its core features: iron dyshomeostasis, lipid peroxidation accumulation, glutathione depletion, and impaired GPX4 activity. The metabolic underpinnings of ferroptosis involve multiple pathways—iron, lipid, amino acid, and glucose metabolism, as well as the mevalonate pathway and key regulatory signals such as p53 and NRF2—all converging to promote oxidative stress, iron overload, and membrane damage.

In diabetic cardiomyopathy and atherosclerosis, ferroptosis contributes to disease initiation and progression via cell type-specific mechanisms, driving myocardial dysfunction, plaque instability, and inflammatory amplification. Targeting ferroptosis with iron chelators, antioxidants, LOX inhibitors, ACSL4 inhibitors, nitroxides, and selenium supplements offers promising therapeutic avenues to mitigate cellular damage and improve clinical outcomes.

However, despite these advances, several unanswered questions remain. The specific mechanistic links between CMDs and systemic metabolism, particularly whether there are common or distinct ferroptosis pathways across different CMDs, require further elucidation. Moreover, it is unclear whether other metabolic or non-metabolic pathways beyond ferroptosis contribute to CMD pathogenesis or could be leveraged for therapy. The translation of ferroptosis research into clinical practice is also hindered by the lack of validated biomarkers and non-invasive diagnostic tools.

Future research should adopt a multidisciplinary approach to explore the shared and unique metabolic vulnerabilities across CMDs, with an emphasis on ferroptosis and beyond. Developing combination therapies that target multiple nodes within the ferroptosis network, alongside other metabolic pathways, may enhance treatment efficacy. Concurrently, efforts to identify and validate ferroptosis-related biomarkers and imaging techniques will be crucial for early diagnosis, risk stratification, and personalized treatment strategies. Integrating mechanistic insights, drug development, and diagnostic innovation will pave the way for breakthroughs in the management of cardiometabolic diseases.

## Figures and Tables

**Table 1:** Representative ferroptosis inhibitors and their mechanisms of action.

Sort	Ferroptosis Inhibitors	Mechanism of Action	References
Iron Chelators	Deferoxamine (DFO)	Chelates Fe^2+^; upregulates GPX4, GSH, and SLC7A11	PMID: 30539824
	Deferiprone (DFP)	Chelates Fe^2+^; inhibits specific histone demethylases	PMID: 30886160
	Deferasirox (DFX)	Chelates Fe^2+^	PMID: 30811439
	Dexrazoxane (DXZ)	Chelates Fe^2+^; upregulates GPX4 and FTH1	PMID: 34336950; PMID: 38232458
	Thymosin β4	Chelates Fe^2+^	PMID: 35008976
	1,10-fenpyrroline	Chelates Fe^2+^	PMID: 30734774
	2,2′-bipyridine	Chelates Fe^2+^	PMID: 30734774
	purine derivatives	Chelates Fe^2+^	PMID: 35188704
Antioxidants	Ferrostain-1	Scavenges lipid ROS; reduces iron levels	PMID: 36081288
	Liproxstain-1	Scavenges lipid ROS; upregulates GPX4 and FSP1	PMID: 34511597; PMID: 36081288
	XJB-5-131	Scavenges mitochondrial ROS	PMID: 35404288
	Maresin1	Reduces ROS; activates NRF2/HO-1/GPX4 pathway	PMID: 35444546
	Naotiaifang (NTE)	Reduces TFR1, DMT1, ROS, iron; upregulates SLC7A11, GPX4, GSH	PMID: 32107172
LOX Inhibitors	Zileuton	Inhibits 5-LOX	PMID: 27380038
	PD146176	Inhibits 15-LOX	PMID: 26040494
	Triacsin C	Broad-spectrum ACSL inhibitor	PMID: 37133631
ACSL4 Inhibitors	Abemaciclib	CDK4/6 inhibitor	PMID: 34510514; PMID: 36872049
	Rosiglitazone	Inhibits ACSL4 expression via PPARγ activation	PMID: 37675456
Nitroxides	Troglitazone	Inhibits ACSL4 via PPARγ activation	PMID: 35869042
	Nuclear enriched transcript 1(NEAT1)	downward revision of ACSL4, SLC7A11,GPX4	PMID: 33730930
	TEMPO	Inhibits Fenton reaction; suppression of hydroxyl radical generation	PMID: 28042034
	Nitric oxide (NO)	Lipid peroxidation inhibition	PMID: 34329739
Selenium Supplements	Selenium	Upregulates GPX4; reduces MDA levels	PMID: 37781125; PMID: 31820398

## Data Availability

No new data were created or analyzed in this study. Data sharing is not applicable to this article.

## Abbreviations

ACSL4	acyl-CoA synthetase long-chain family member 4
AS	atherosclerosis
DCM	diabetic cardiomyopathy
DMT1	divalent metal transporter 1
FSP1	ferroptosis suppressor protein 1
GPX4	glutathione peroxidase 4
GSH	glutathione (reduced)
HO-1	heme oxygenase 1
LOX	lipoxygenase
LPCAT3	lysophosphatidylcholine acyltransferase 3
LIP	labile iron pool
MUFA	monounsaturated fatty acid
NRF2	nuclear factor erythroid 2-related factor 2
NTBI	non-transferrin-bound iron
ox-LDL	oxidized low-density lipoprotein
PUFA	polyunsaturated fatty acid
PUFA-PL	polyunsaturated fatty acid-containing phospholipid
RCD	regulated cell death
SLC7A11	solute carrier family 7 member 11
SLC40A1	solute carrier family 40 member 1 (Ferroportin)
STEAP3	six-transmembrane epithelial antigen of prostate 3
TF	transferrin
TFR1	transferrin receptor 1
VSMC	vascular smooth muscle cell
